# Amyloid β Modification: A Key to the Sporadic Alzheimer's Disease?

**DOI:** 10.3389/fgene.2017.00058

**Published:** 2017-05-15

**Authors:** Evgeny P. Barykin, Vladimir A. Mitkevich, Sergey A. Kozin, Alexander A. Makarov

**Affiliations:** Engelhardt Institute of Molecular Biology, Russian Academy of SciencesMoscow, Russia

**Keywords:** beta amyloid, post-translational Modifications (PTM), proteostasis, Sporadic Alzheimer's disease, proteinopathies, Aging

Last year marked 25 years of research into the amyloid hypothesis of Alzheimer's disease (AD) (Selkoe and Hardy, 2016). Over the last few years, studies on this subject have provided a number of insights into the pathology of the most widespread cognitive disorder of aging; however, a successful treatment strategy has yet to be developed. The amyloid hypothesis was on the edge of being discredited due to the indistinct correlation between β-amyloid (Aβ) deposition and neuronal loss (Holmes et al., 2008; Mullane and Williams, 2013). However, recent studies have defended the Aβ peptide as a causative factor in AD and have proved it to be necessary but not sufficient to explain the pathogenesis of the disease in full (Musiek and Holtzman, 2015). An updated hypothesis suggests that, Aβ accumulation is an essential trigger that initiates a pathological cascade implicating tau protein, synuclein, and other aggregation-prone proteins. The questions still to be answered are: *which events* pull the trigger on the Aβ aggregation cascade and *how exactly* does destabilization of amyloid proteostasis promote the downstream tau pathology. The answer to the first question is clear and transparent in familial AD (fAD) as it is induced by genetic aberrations. However, it remains a mystery in so-called sporadic AD (sAD), which accounts for more than 90% of the disease cases. Currently, sporadic AD is a major subject of study with the primary focus being, to make it “less sporadic” by finding a genetic or aging-related basis for the disease.

A possible insight into the problem of sAD was found within the amyloid plaques. An analysis of plaque composition has shown that aggregated β-amyloid peptides are modified in different ways, primarily by isomerization and truncation of Aβ (Roher et al., 1993). Subsequent *in vitro* and *in vivo* studies revealed that a plethora of modifications exhibit pathogenic features; these include: increased aggregation, neurotoxicity, amyloidogenicity, and an ability to suppress long-term potentiation in the hippocampus (Shimizu et al., 2002; Kumar, 2011; Al-Hilaly et al., 2013; Kozin S. et al., 2013; Mitkevich et al., 2013; Barykin et al., 2016).

Hence, we propose a model in which, aberrant post-translational modification (PTM) of the amyloid β peptide increases amyloid neurotoxicity and facilitates its aggregation thus initiating or promoting progression of sAD.

## Aβ peptide: from intact to modified

Amyloid β modification is a complex process that occurs both enzymatically and non-enzymatically. Many proteins are already shown to interact with Aβ leading to alteration of its structure or repair of pathogenic modifications. However, for many modified Aβ species, purified from AD brain tissue, the source of origin remains unknown (Kummer and Heneka, 2014).

**Figure 1:**
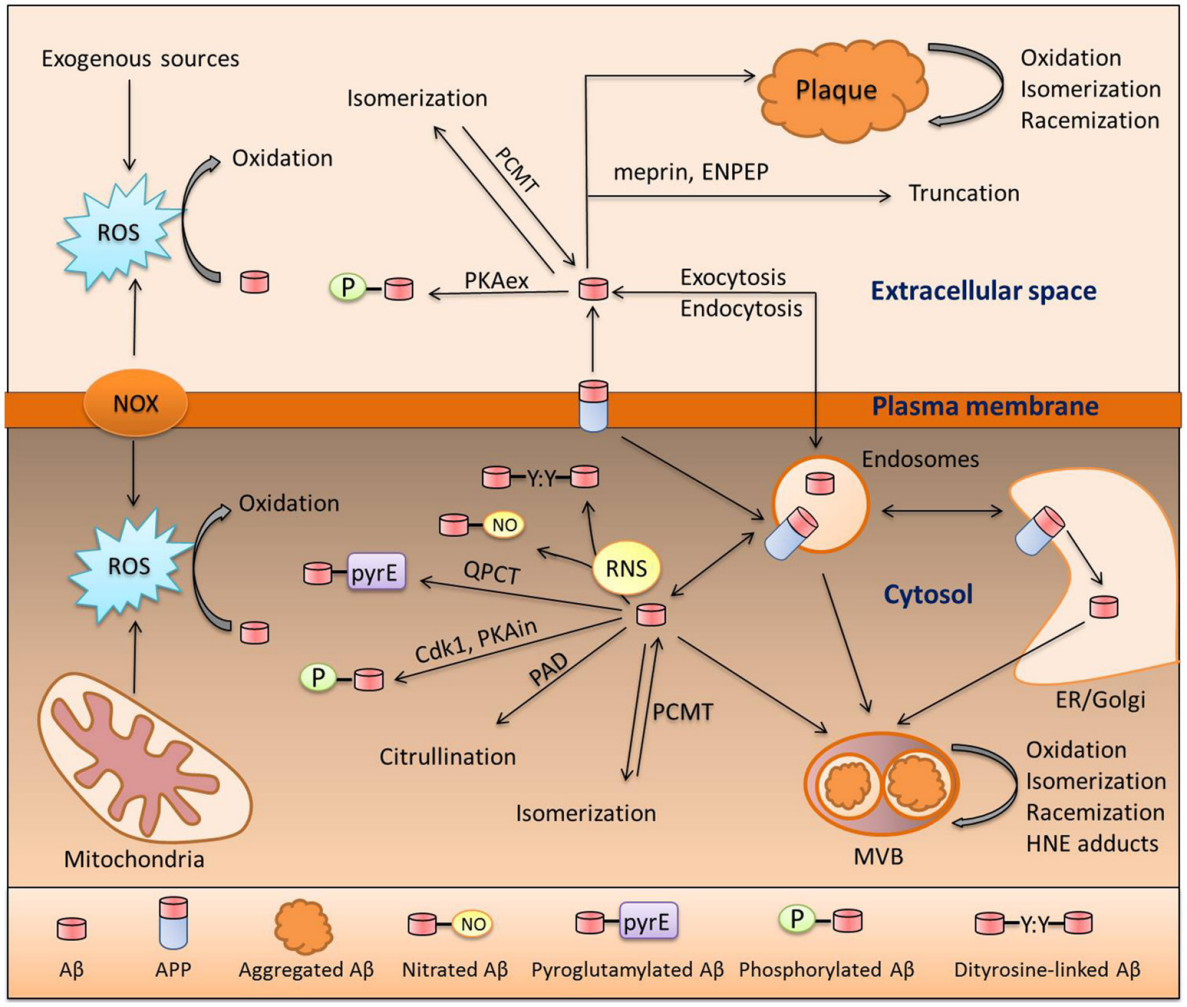
**Pathways of Aβ modification**. Aβ is a product of amyloid precursor protein (APP) cleavage at the plasma membrane or inside the cell in the endosomal compartment or ER/Golgi. These two pools exchange Aβ via endo- and exocytosis. Aβ in both of these pools can undergo oxidation due to interaction with reactive oxygen species (ROS) produced by NADPH-oxidase (NOX), the mitochondrial respiratory chain, or exogenous sources. Reactive nitrogen species (RNS) produced by nitric oxide synthase (NOS) isoforms also interact with Aβ which, results in nitration of the Tyr10 residue or formation of covalently linked dimers of Aβ. Extracellular Aβ is phosphorylated by extracellular protein kinase A (PKAex) and intracellular Aβ is subjected to phosphorylation by both intracellular PKA (PKAin) and cdc2 kinase (Cdk1). An exclusive modification of intracellular Aβ is citrullination by peptidyl arginyl deiminase (PAD). Aspartic residues of Aβ are prone to spontaneous isomerization or racemization, and this isomerization can be reversed by protein carboxyl methyltransferase 1 (PCMT1). In amyloid deposits (plaques or multivesicular bodies [MVB]), Aβ undergoes oxidative damage which leads to the formation of adducts with 4-hydroxynonenal (HNE); a product of lipid peroxidation. Aminopeptidase A (ENPEP) and meprin can truncate Aβ. Lastly, amyloid beta can be pyroglutamylated at the E3 and E11 sites by glutaminyl-peptide cyclotransferase (QPCT).

Prior to discussing the role of modified Aβ in sAD, we will first focus on the life cycle of the Aβ molecule from its formation to its degradation or aggregation; taking into consideration, all modifications along the way. Beta-amyloid is produced via proteolytic cleavage of APP protein by beta-secretase (BACE) and gamma-secretase (Huang and Mucke, 2012); this is termed the amyloidogenic pathway. The non-amyloidogenic pathway is mediated by alpha-secretase (ADAM10). It is important to mention that cleavage by gamma-secretase is imprecise and results in production of an Aβ peptide ranging from 37 to 43 amino acids in length; notably the 42 residue species is considered to be the most pathogenic (Haass and Selkoe, 2007). Production of Aβ may occur at three different sites: on the plasma membrane, in the ER/Golgi or in endocytic vesicles. This decision helps determine its fate and defines the set of possible modifications that can be made to the peptide, as different Aβ-modifying enzymes are assigned to specific cellular compartments (Hartmann et al., 1997; Thinakaran and Koo, 2008). In Figure 1 below, we present a putative scheme for the amyloid peptide modification process inside and outside of the cell and both in solution and as aggregates (Figure 1). Some of the modifications present are enzymatic, some are triggered by low-molecular compounds such as peroxynitrite or 4-hydroxynonenal (HNE), and two of them are spontaneous, namely racemization and isomerization. Formation of N-truncated amyloid has not been well studied, however it is possible that it originates from proteolytic cleavage by aminopeptidase A (ENPEP), meprin or BACE or alternatively via non-enzymatic hydrolysis of peptide bonds (Kummer and Heneka, 2014). A huge body of evidence supports the pathogenic role of individual Aβ modifications; however, no research has been done to investigate the orchestrated action of different modifications on a single molecule of amyloid peptide. Additive or synergistic effects of such modifications may potentially increase the pathogenic properties of Aβ peptide far above the level of the widely studied intact Aβ. These modifications can promote accumulation of amyloid and plaque formation as they hamper its clearance and increase aggregation (Kumar, 2011; Kozin S. A. et al., 2013). Another blind spot in the studies of Aβ PTM is its connection with tau pathology. Tau hyperphosphorylation (HP) is presumably induced by Aβ, leading to systemic brain pathology (Oddo et al., 2006) and this transition might be caused by modified Aβ peptides. However, the association of HP-tau and Aβ modifications was only studied and observed for pyroglutamylated amyloid peptide (Mandler et al., 2014). We propose that studies of the relationship between Aβ PTM and tau pathology may contribute substantially to the understanding of AD development.

## Aging interferes with Aβ modification

The principal fact that drew our attention to amyloid PTMs as a presumable cause of sAD, is that the PTM process is disrupted with aging. It was shown both directly in studies where the accumulation of modified proteins was measured (Levine and Stadtman, 2001), and indirectly as we know that proteostasis itself is disturbed in the aged body (Dubnikov and Cohen, 2015; Labbadia and Morimoto, 2015). It is known that reactive oxygen species (ROS) production and neutralization is destabilized in the aged body due to elevation of NOX activity (Dasuri et al., 2013) and an increase in mitochondrial respiratory chain leakage that is accompanied by an accumulation of mutations in mitochondrial DNA (Bratic and Larsson, 2013). HNE is a product of lipid peroxidation and its production increases with aging as a side effect of chronic oxidative stress (Castro et al., 2016). It has also been shown that nitric oxide synthase is upregulated in AD; however, it is not clear whether it is a normal part of the aging process or a pathological event (Domek-Łopacińska and Strosznajder, 2010). Meanwhile, isomerized and deaminated proteins have a tendency to accumulate naturally in an aging organism, and in carboxyl methyltransferase-deficient mice damaged proteins have also been shown to accumulate in the brain (Kim et al., 1997; Clarke, 2003). The phosphorylation process is regulated by balancing kinase and phosphatase activity and is also disrupted with aging (Magnoni et al., 1991; Rajagopal et al., 2016; Thomas and Haberman, 2016). Citrullination is another modification that is known to increase in aged body (Osaki and Hiramatsu, 2016). The important point is that modifications can create pathogenic networks with a positive feedback. It was shown that ROS-induce dityrosine crosslinking of Aβ results in formation of stable and poorly degradable oligomers (Al-Hilaly et al., 2013). Aβ increases ROS production (Butterfield and Swomley, 2012), which then promotes further inhibition of the amyloid clearance system and may result in a positive feedback-driven cascade of accumulation; such a cascade has already been shown for the Aβ and HNE interaction (Ellis et al., 2010). Taken together, all of these findings make it probable that disturbance of Aβ modification processes with age leads to the rise of different pathogenic processes including AD.

## Hereditary variance of amyloid PTMs

Since we propose an aging-related disturbance in the Aβ modification process as a cause of AD, one may ask whether every aging individual is destined to suffer from Aβ accumulation, AD, and a resulting steady cognitive decline with age. This does not happen and AD obviously requires additional triggers besides senescence-related pathogenic modification of Aβ peptides. These triggers are widely discussed and a plethora of work has been conducted to identify AD risk factors. The risk factors identified include: smoking, sleep deprivation, brain trauma, diabetes, bacterial infections, viral infections, and gut microbiota alteration (Itzhaki et al., 1997; Miklossy, 2008; Kang et al., 2009; Naseer et al., 2014; Reitz and Mayeux, 2014). The most studied trigger of AD is genetic background. According to estimations, based on human pedigree analysis, up to 80% of all Alzheimer's cases are hereditary (Bergem, 1994). The first genes that were identified as genes in which mutations lead to fAD were: APP, BACE, and gamma-secretase genes, called PSEN1 and PSEN2. They are responsible for most cases of familial AD and can dramatically increase overall Aβ production or shift the production ratio in favor of Aβ 1-42 (Bertram et al., 2010). Increased Aβ burden results in early amyloid accumulation and this in turn leads to an early-onset development of brain pathology (Huang and Mucke, 2012). However, fAD only accounts for about 1% of registered AD cases and late-onset AD (LOAD) or sAD is more or less beyond prediction (Campion et al., 1999; Bertram et al., 2010). In sAD, ApoE gene variants were associated with an increased risk of disease and could account for up to 20% of LOAD cases (Ertekin-Taner, 2010). However, to date the efforts to identify other sAD-modifying genes with a comparable magnitude of influence have been unsuccessful and we suggest that future work should focus on the genetics of Aβ-modifying enzymes. Currently an association between genetic variants of Aβ-modifying enzymes and AD is only shown for NOS2 (Akomolafe et al., 2006) and QPCT (Saykin et al., 2010), however for the latter the association does not have genome-wide significance. It is very possible that the lack of such associations is due to the nature of the tool that was used in prior studies to identify disease-modifying genes. The primary tool for such investigations is genome-wide association studies (GWAS), which have brought many gene-disease associations to our attention over the years (Singleton and Hardy, 2016). However, GWAS usually lacks full-genome coverage and fails to detect statistically significant associations with small effects (Naj et al., 2017). GWAS findings are dependent on a chosen cohort and many candidate genes are often thrown away. GWAS associations do not permit an inference of causation (Naj et al., 2017), so the role of individual genetic studies based on additional data is not diminished. Most enzymes featured in Figure 1 are already associated with other genetic pathologies, including neurological diseases; ENPEP is associated with Koch Hypertension (Kato et al., 2011); QPCT is associated with schizophrenia and frontotemporal dementia (Zhang et al., 2016); and PCMT1 with premature ovarian failure (Pyun et al., 2009). Variants of these may likewise be important for the development of AD. Such guidance may facilitate further genetic studies taking into consideration the potential synergy between the impairment of different modifications.

Aβ modifications and genetic alterations is a vast, yet poorly studied field with the potential to contribute substantially to the understanding of AD pathogenesis. The modification process results in the formation of pathogenic Aβ species, the level of which may increase with age and due to hereditary factors. To summarize, we hypothesize that modification of Aβ is a major contributor to sAD and targeting of the modified peptides or modification enzymes could serve as a novel therapeutic mechanism or provide a new means of diagnosis.

## Author contributions

All authors listed, have made substantial, direct and intellectual contribution to the work, and approved it for publication.

## Funding

The study was funded by the Russian Science Foundation (grant #14-24-00100).

### Conflict of interest statement

The authors declare that the research was conducted in the absence of any commercial or financial relationships that could be construed as a potential conflict of interest.

## References

[B1] Akomolafe A. Lunetta K. Erlich P. Cupples L. Baldwin C. Huyck M.. (2006). Genetic association between endothelial nitric oxide synthase and Alzheimer disease. Clin. Genet. 70, 49–56. 10.1111/j.1399-0004.2006.00638.x 16813604

[B2] Al-Hilaly Y. K. Williams T. L. Stewart-Parker M. Ford L. Skaria E. Cole M.. (2013). A central role for dityrosine crosslinking of Amyloid-β in Alzheimer's disease. Acta Neuropathol. Commun. 1:83. 10.1186/2051-5960-1-83 24351276 PMC3880074

[B3] Barykin E. P. Petrushanko I. Y. Burnysheva K. M. Makarov A. A. Mitkevich V. A. (2016). [Isomerization of Asp7 increases the toxic effects of amyloid beta and its phosphorylated form in SH-SY5Y neuroblastoma cells]. Mol. Biol. Mosk. 50, 863–869. 10.7868/S0026898416050037 27830689

[B4] Bergem A. L. M. (1994). Heredity in dementia of the Alzheimer type. Clin. Genet. 46, 144–149. 10.1111/j.1399-0004.1994.tb04216.x 7988071

[B5] Bertram L. Lill C. M. Tanzi R. E. (2010). The Genetics of Alzheimer Disease: back to the future. Neuron 68, 270–281. 10.1016/j.neuron.2010.10.013 20955934

[B6] Bratic A. Larsson N.-G. (2013). The role of mitochondria in aging. J. Clin. Invest. 123, 951–957. 10.1172/JCI64125 23454757 PMC3582127

[B7] Butterfield D. A. Swomley A. M. (2012). Amyloid β-Peptide (1–42)-Induced Oxidative Stress in Alzheimer Disease: importance in Disease Pathogenesis and Progression. Antioxid. Redox Signal. 19, 823–835. 10.1089/ars.2012.5027 23249141 PMC3749710

[B8] Campion D. Dumanchin C. Hannequin D. Dubois B. Belliard S. Puel M.. (1999). Early-onset autosomal dominant Alzheimer disease: prevalence, genetic heterogeneity, and mutation spectrum. Am. J. Hum. Genet. 65, 664–670. 10.1086/302553 10441572 PMC1377972

[B9] Castro J. P. Jung T. Grune T. Siems W. (2016). 4-Hydroxynonenal (HNE) modified proteins in metabolic diseases. Free Radic. Biol. Med. [Epub ahead of print]. 10.1016/j.freeradbiomed.2016.10.497 27815191

[B10] Clarke S. (2003). Aging as war between chemical and biochemical processes: protein methylation and the recognition of age-damaged proteins for repair. Ageing Res. Rev. 2, 263–285. 10.1016/S.1568-1637(03)00011-4 12726775

[B11] Dasuri K. Zhang L. Keller J. N. (2013). Oxidative stress, neurodegeneration, and the balance of protein degradation and protein synthesis. Free Radic. Biol. Med. 62, 170–185. 10.1016/j.freeradbiomed.2012.09.016 23000246

[B12] Domek-Łopacińska K. U. Strosznajder J. B. (2010). Cyclic GMP and Nitric Oxide Synthase in Aging and Alzheimer's Disease. Mol. Neurobiol. 41, 129–137. 10.1007/s12035-010-8104-x 20213343

[B13] Dubnikov T. Cohen E. (2015). Proteostasis collapse, inter-tissue communication, and the regulation of aging at the organismal level. Front. Genet. 6:80. 10.3389/fgene.2015.00080 25798145 PMC4350417

[B14] Ellis G. Fang E. Maheshwari M. Roltsch E. Holcomb L. Zimmer D.. (2010). Lipid oxidation and modification of amyloid-β (Aβ) *in vitro* and *in vivo*. J. Alzheimers Dis. 22, 593–607. 10.3233/JAD-2010-100960 20847409

[B15] Ertekin-Taner N. (2010). Genetics of Alzheimer disease in the pre- and post-GWAS era. Alzheimers Res. Ther. 2:3. 10.1186/alzrt26 20236449 PMC2874262

[B16] Haass C. Selkoe D. J. (2007). Soluble protein oligomers in neurodegeneration: lessons from the Alzheimer's amyloid beta-peptide. Nat. Rev. Mol. Cell Biol. 8, 101–112. 10.1038/nrm2101 17245412

[B17] Hartmann T. Bieger S. C. Brühl B. Tienari P. J. Ida N. Allsop D. (1997). Distinct sites of intracellular production for Alzheimer's disease Aβ40/42 amyloid peptides. Nat. Med. 3, 1016–1020. 10.1038/nm0997-1016 9288729

[B18] Holmes C. Boche D. Wilkinson D. Yadegarfar G. Hopkins V. Bayer A.. (2008). Long-term effects of Aβ42 immunisation in Alzheimer's disease: follow-up of a randomised, placebo-controlled phase I trial. Lancet 372, 216–223. 10.1016/S0140-6736(08)61075-2 18640458

[B19] Huang Y. Mucke L. (2012). Alzheimer Mechanisms and Therapeutic Strategies. Cell 148, 1204–1222. 10.1016/j.cell,0.2012.02.040 22424230 PMC3319071

[B20] Itzhaki R. F. Lin W.-R. Shang D. Wilcock G. K. Faragher B. Jamieson G. A. (1997). Herpes simplex virus type 1 in brain and risk of Alzheimer's disease. Lancet 349, 241–244. 10.1016/S0140-6736(96)10149-5 9014911

[B21] Kang J.-E. Lim M. M. Bateman R. J. Lee J. J. Smyth L. P. Cirrito J. R. (2009). Amyloid-β dynamics are regulated by orexin and the sleep-wake cycle. Science 326, 1005–1007. 10.1126/science.1180962 19779148 PMC2789838

[B22] Kato N. Takeuchi F. Tabara Y. Kelly T. N. Go M. J. Sim X.. (2011). Meta-analysis of genome-wide association studies identifies common variants associated with blood pressure variation in east Asians. Nat. Genet. 43, 531–538. 10.1038/ng.834 21572416 PMC3158568

[B23] Kim E. Lowenson J. D. MacLaren D. C. Clarke S. Young S. G. (1997). Deficiency of a protein-repair enzyme results in the accumulation of altered proteins, retardation of growth, and fatal seizures in mice. Proc. Natl. Acad. Sci. U.S.A. 94, 6132–6137. 10.1073/pnas.94.12.6132 9177182 PMC21014

[B24] Kozin S. A. Cheglakov I. B. Ovsepyan A. A. Telegin G. B. Tsvetkov P. O. Lisitsa A. V.. (2013). Peripherally Applied Synthetic Peptide isoAsp7-Aβ(1-42) Triggers Cerebral β-Amyloidosis. Neurotox. Res. 24, 370–376. 10.1007/s12640-013-9399-y 23670398

[B25] Kozin S. Cheglakov I. Ovsepyan A. Telegin G. Tsvetkov P. Lisitsa A. (2013). Amyloidogenic properties of isoAsp7-containing beta-amyloid peptide. Alzheimers Dement. J. Alzheimers Assoc. 9:P857 10.1016/j.jalz.2013.08.176

[B26] Kumar S. (2011). Phosphorylation of amyloid beta (Aβ) peptides – A trigger for formation of toxic aggregates in Alzheimer's disease. Aging 3, 803–812. 10.18632/aging.100362 21869458 PMC3184981

[B27] Kummer M. P. Heneka M. T. (2014). Truncated and modified amyloid-beta species. Alzheimers Res. Ther. 6:28. 10.1186/alzrt258 25031638 PMC4055046

[B28] Labbadia J. Morimoto R. I. (2015). The Biology of Proteostasis in Aging and Disease. Annu. Rev. Biochem. 84, 435–464. 10.1146/annurev-biochem-060614-033955 25784053 PMC4539002

[B29] Levine R. L. Stadtman E. R. (2001). Oxidative modification of proteins during aging. Exp. Gerontol. 36, 1495–1502. 10.1016/S0531-5565(01)00135-8 11525872

[B30] Magnoni M. S. Govoni S. Battaini F. Trabucchi M. (1991). The aging brain: protein phosphorylation as a target of changes in neuronal function. Life Sci. 48, 373–385. 10.1016/0024-3205(91)90492-T 1671520

[B31] Mandler M. Walker L. Santic R. Hanson P. Upadhaya A. R. Colloby S. J.. (2014). Pyroglutamylated amyloid-β is associated with hyperphosphorylated tau and severity of Alzheimer's disease. Acta Neuropathol. (Berl.) 128, 67–79. 10.1007/s00401-014-1296-9 24861310

[B32] Miklossy J. (2008). Chronic Inflammation and Amyloidogenesis in Alzheimer's Disease – Role of Spirochetes. J. Alzheimers Dis. 13, 381–391. 10.3233/JAD-2008-13404 18487847

[B33] Mitkevich V. A. Petrushanko I. Y. Yegorov Y. E. Simonenko O. V. Vishnyakova K. S. Kulikova A. A.. (2013). Isomerization of Asp7 leads to increased toxic effect of amyloid-beta42 on human neuronal cells. Cell Death Dis 4:e939. 10.1038/cddis.2013.492 24287700 PMC3847340

[B34] Mullane K. Williams M. (2013). Alzheimer's therapeutics: continued clinical failures question the validity of the amyloid hypothesis—but what lies beyond? Biochem. Pharmacol. 85, 289–305. 10.1016/j.bcp.2012.11.014 23178653

[B35] Musiek E. S. Holtzman D. M. (2015). Three dimensions of the amyloid hypothesis: time, space, and “wingmen.” Nat. Neurosci. 18, 800–806. 10.1038/nn.4018 26007213 PMC4445458

[B36] Naj A. C. Schellenberg G. D. for the Alzheimer's Disease Genetics Consortium (2017). Genomic variants, genes, and pathways of Alzheimer's disease: an overview. Am. J. Med. Genet. B Neuropsychiatr. Genet. 174, 5–26. 10.1002/ajmg.b.32499 27943641 PMC6179157

[B37] Naseer M. I. Bibi F. Alqahtani M. H. Chaudhary A. G. Azhar E. I. Kamal M. A.. (2014). Role of gut microbiota in obesity, Type 2 diabetes and Alzheimer's Disease. CNS Neurol. Disord. Drug Targets 13, 305–311. 10.2174/18715273113126660147 24059313

[B38] Oddo S. Caccamo A. Tran L. Lambert M. P. Glabe C. G. Klein W. L.. (2006). Temporal Profile of Amyloid-β (Aβ) Oligomerization in an *in vivo* Model of Alzheimer Disease A LINK BETWEEN Aβ AND TAU PATHOLOGY. J. Biol. Chem. 281, 1599–1604. 10.1074/jbc.M507892200 16282321

[B39] Osaki D. Hiramatsu H. (2016). Citrullination and deamidation affect aggregation properties of amyloid β -proteins. Amyloid 23, 234–241. 10.1080/13506129.2016.1240076 27791396

[B40] Pyun J.-A. Kang H. Lee S. K. Kim M. Kwack K. (2009). Association between polymorphisms in the protein L-isoaspartate (D-aspartate) O-methyltransferase gene and premature ovarian failure. Fertil. Steril. 91, 1362–1365. 10.1016/j.fertnstert.2008.03.078 18582870

[B41] Rajagopal S. Deb I. Poddar R. Paul S. (2016). Aging is associated with dimerization and inactivation of the brain-enriched tyrosine phosphatase STEP. Neurobiol. Aging 41, 25–38. 10.1016/j.neurobiolaging.2016.02.004 27103516 PMC4841919

[B42] Reitz C. Mayeux R. (2014). Alzheimer disease: epidemiology, diagnostic criteria, risk factors and biomarkers. Biochem. Pharmacol. 88, 640–651. 10.1016/j.bcp.2013.12.024 24398425 PMC3992261

[B43] Roher A. E. Lowenson J. D. Clarke S. Wolkow C. Wang R. Cotter R. J.. (1993). Structural alterations in the peptide backbone of beta-amyloid core protein may account for its deposition and stability in Alzheimer's disease. J. Biol. Chem. 268, 3072–3083. 8428986

[B44] Saykin A. J. Shen L. Foroud T. M. Potkin S. G. Swaminathan S. Kim S.. (2010). Alzheimer's Disease Neuroimaging Initiative biomarkers as quantitative phenotypes: genetics core aims, progress, and plans. Alzheimers Dement. 6, 265–273. 10.1016/j.jalz.2010.03.013 20451875 PMC2868595

[B45] Selkoe D. J. Hardy J. (2016). The amyloid hypothesis of Alzheimer's disease at 25 years. EMBO Mol. Med. 8, 595–608. 10.15252/emmm.201606210 27025652 PMC4888851

[B46] Shimizu T. Fukuda H. Murayama S. Izumiyama N. Shirasawa T. (2002). Isoaspartate formation at position 23 of amyloid beta peptide enhanced fibril formation and deposited onto senile plaques and vascular amyloids in Alzheimer's disease. J. Neurosci. Res. 70, 451–461. 10.1002/jnr.10350 12391606

[B47] Singleton A. Hardy J. (2016). The evolution of genetics: Alzheimer's and Parkinson's Diseases. Neuron 90, 1154–1163. 10.1016/j.neuron.2016.05.040 27311081 PMC4936485

[B48] Thinakaran G. Koo E. H. (2008). Amyloid precursor protein trafficking, processing, and function. J. Biol. Chem. 283, 29615–29619. 10.1074/jbc.R800019200 18650430 PMC2573065

[B49] Thomas A. Haberman A. (2016). Phosphorylation of Neuronal Proteins in Drosophila melanogaster Changes With Age. FASEB J. 30, 1080.3–1080.3.

[B50] Zhang Q.-Q. Jiang T. Gu L.-Z. Zhu X.-C. Zhao H.-D. Gao Q.. (2016). Common Polymorphisms Within QPCT Gene Are Associated with the Susceptibility of Schizophrenia in a Han Chinese Population. Mol. Neurobiol. 53, 6362–6366. 10.1007/s12035-015-9541-3 26572640

